# Estimation of the influenza‐associated respiratory hospitalization burden using sentinel surveillance data, Lebanon, 2015–2020

**DOI:** 10.1111/irv.13138

**Published:** 2023-04-23

**Authors:** Zeina Farah, Hala Abou El Naja, Stefano Tempia, Nadine Saleh, Abdinasir Abubakar, Patrick Maison, Nada Ghosn

**Affiliations:** ^1^ Epidemiological Surveillance Program Ministry of Public Health Beirut Lebanon; ^2^ Eastern Mediterranean Regional Office World Health Organization Cairo Egypt; ^3^ Global Influenza Program World Health Organization Geneva Switzerland; ^4^ Faculty of Public Health Lebanese University Beirut Lebanon; ^5^ INSPECT‐LB (Institut National de Santé Publique, d'Épidémiologie Clinique et de Toxicologie) Beirut Lebanon; ^6^ Country office World Health Organization Beirut Lebanon; ^7^ French National Agency for Medicines and Health Products Safety (ANSM) Saint‐Denis France; ^8^ Paris‐Est Creteil University Creteil France

**Keywords:** global burden of disease, hospitalization, human, influenza, Lebanon

## Abstract

**Introduction:**

Influenza epidemics cause around 3 to 5 million cases of severe illness worldwide every year. Estimates are needed for a better understanding of the burden of disease especially in low‐ and middle‐income countries. The objective of this study is to estimate the number and rate of influenza‐associated respiratory hospitalizations in Lebanon during five influenza seasons (2015–2016 to 2019–2020) by age and province of residence in addition to estimating the influenza burden by level of severity.

**Methods:**

The severe acute respiratory infection sentinel surveillance system was used to compute influenza positivity from the influenza laboratory confirmed cases. The total of respiratory hospitalizations under the influenza and pneumonia diagnosis was retrieved from the Ministry of Public Health hospital billing database. Age‐specific and province‐specific frequencies and rates were estimated for each season. Rates per 100 000 population were calculated with 95% confidence levels.

**Results:**

The estimated seasonal average of influenza‐associated hospital admission was 2866 for a rate of 48.1 (95% CI: 46.4–49.9) per 100 000. As for the distribution by age group, the highest rates were seen in the two age groups ≥65 years and 0–4 years whereas the lowest rate was for the age group 15–49 years. For the distribution by province of residence, the highest influenza‐associated hospitalization rates were reported from the Bekaa‐Baalback/Hermel provinces.

**Conclusion:**

This study shows the substantial burden of influenza in Lebanon mainly on high‐risk groups (≥65 years and <5 years). It is crucial to translate these findings into policies and practices to reduce the burden and estimate the illness‐related expenditure and indirect costs.

## 1 INTRODUCTION

According to the World Health Organization (WHO), the seasonal influenza epidemics cause annually around 3 to 5 million cases of severe illness and around 290 000 to 650 000 respiratory deaths mainly among high risk groups like older adults and children less than 5 years of age^1^ causing, as a result, a significant economic burden. There is also a considerable economic impact of influenza among other age groups presenting with mild to moderate clinical symptoms. This is due to the loss or decrease in productivity associated with the increase in worker absenteeism.^2^

Nonpharmaceutical public health measures like social distancing, hand hygiene, and cough/sneezing etiquette are important for preventing respiratory diseases; however, vaccination remains the most important intervention for influenza prevention.^3^ The Strategic Advisory Group of Experts on Immunization (SAGE) recommends influenza vaccination to certain high‐risk groups to be administered each year to reduce the risk of serious complications that could lead to hospitalization and death. However, the influenza vaccine uptake remains low in several countries including countries of the WHO Eastern Mediterranean Region.^4^

Therefore, it is critical to estimate the burden of disease to better understand the impact of the disease especially in vulnerable groups like young children and older adults.^5^ This improved understanding of influenza disease burden also assist to inform evidence‐based policies and decisions when allocating limited resources and planning preventive measures to limit the influenza transmission and reduce the costs thereof.^6^ Burden of disease studies also enhance surveillance and analytical capacities of countries to be used during pandemics.^7^

Some studies have been conducted worldwide to estimate the burden of influenza. Different approaches have been used to estimate the influenza burden and the different sections of the disease severity pyramid. Used methods depend on the available data sources and the type of calculated estimates. According to the available results, the highest influenza‐associated hospital admission rates were reported among children less than 5 years and older adults ≥65 years but the effect of influenza on the other age groups could not be ignored.^8^, ^9^, ^10^, ^11^

Although, as per WHO, global estimates are needed for a better understanding of the burden of disease worldwide,^7^ there is a paucity of reliable and up‐to‐date estimates on the burden of influenza at the global and regional levels especially in low and middle‐income countries^12^ where the impact of influenza is expected to be the highest.^7^ Similarly, no such estimates are available in Lebanon, which is a country in the Eastern Mediterranean region with a population of 5 to 6 million inhabitants, based on the United Nations Population division (UNPD) data.

We aimed in this study to estimate the number and rate of influenza‐associated respiratory hospitalization in Lebanon during five influenza seasons (2015–2016 to 2019–2020) by age and province of residence in addition to estimate the influenza burden by level of severity.

## 2 METHODS

### 2.1 Data sources

The main data sources used in this study were the severe acute respiratory infections (SARI) sentinel surveillance system and the MoPH hospital billing database.

The SARI sentinel surveillance system launched in 2015 by the Epidemiological Surveillance Unit (ESU) at the Lebanese Ministry of Public Health (MoPH) in collaboration with the WHO aims mainly to estimate morbidity of SARI in Lebanon, identify baseline figures and alert/outbreak thresholds, identify circulating influenza strains, and detect novel viruses. SARI cases were defined as acute respiratory infection patients with a history of fever or measured fever of ≥38°C and cough with onset of symptoms within the last 10 days and requiring hospitalization.^2^ Between 2015 and 2017, data and nasopharyngeal specimens were collected for patients fitting the case definition from 11 sentinel sites. In each province, two or three hospitals were selected from the public and private sectors to ensure representativeness of the data. The number of active sites dropped to 9 in 2018 and 8 in 2019 and 2020. Specimens were tested at the National Influenza Center (NIC) using the Reverse Transcriptase Polymerase Chain Reaction (RT‐PCR) laboratory technique, and results were shared with WHO surveillance platforms like Global Influenza Surveillance and Response System (GISRS). As the sentinel surveillance system was properly functioning between 2015 and 2020, before COVID‐19 pandemic struck and completeness of all the surveillance data including the SARI surveillance completeness was affected, data between 2015 and 2020 were included in the study.

The second source of data used in this study was the MoPH hospital billing database, which includes data on all Lebanese patients admitted to hospitals with different diagnoses and covered by the MoPH. The International Classification of Diseases, Tenth Revision (ICD‐10), coding system is used to code cases' discharge diagnoses in the database. J00‐J99 codes refer to all diseases of the respiratory system while J09‐J18 are used for influenza and pneumonia.^13^ Of note, MoPH covers hospital bills for Lebanese who are not insured by any type of insurance and who are estimated to represent 52.3% of hospitalized Lebanese patients.^14^, ^15^

As for the population estimates, they were based on the UNPD figures stratified by age and year.

### 2.2 Data preparation

Methodological analysis and calculations were based on WHO guidelines.^6^ Data were analyzed for all seasons for the period between week 40 of 2015 and week 39 of 2020. The period between week 40 of 1 year and week 39 of the following year was considered one season. This window was selected based on findings of the influenza severity assessment conducted in Lebanon using the Pandemic Influenza Severity Assessment (PISA) tool.^16^ Cases with missing age, date, and PCR result were excluded from the analysis. The following age groups were considered: 0–4, 5–14, 15–49, 50–64, and ≥65 years. Surveillance data on counts of SARI cases and laboratory‐confirmed influenza were extracted from the SARI sentinel system and stratified by age group. The age‐stratified influenza positivity percentage was then calculated for each season of the studied period by dividing the number of influenza positive specimens by the total SARI tested specimens. Data on total hospitalization from the MoPH hospital billing database were requested under ICD‐10 principal diagnosis codes for influenza/pneumonia (J09‐J18). Cases with missing age were excluded from the analysis. Data were stratified by age and adjusted for MoPH hospital coverage to obtain the estimate of the total number of influenza/pneumonia associated hospitalizations in Lebanon for each season stratified by age group. The hospital billing database covers 52.3% of Lebanese patients,^14^ and based on UNPD estimates and UNHCR data, we estimated that on average Lebanese accounted for 83% of the total population during the study period.^15^, ^17^ Data were adjusted for nationality because the MoPH hospital billing database used for the calculations only includes data for Lebanese patients.

Sensitivity and specificity of the SARI case definition were retrieved from WHO guidelines. Data cleaning and analysis were conducted using Microsoft Excel 2016, QGIS 3.24.3, R software version 4.2.2 and R studio version 2022.2.3.492.

### 2.3 Computed indicators

The following indicators were computed: The estimates of the influenza‐associated respiratory hospitalizations and rates by age group and province of residence during the study period in addition to the seasonal average for each estimate. Distribution of influenza cases and rates by level of severity was also estimated.

### 2.4 Estimation of influenza‐associated respiratory hospitalizations and rates

Descriptive statistical methods were used to estimate influenza‐attributable hospitalizations and rates. As sentinel surveillance sites were distributed throughout the country and the same case definitions and sampling guidelines were used, the catchment population of the sentinel sites was considered representative of the Lebanese population. Therefore, data from the different sentinel sites were pooled to compute a national disease burden estimate.^6^

Age‐specific frequencies and rates were estimated for each season. Rates per 100 000 population were calculated with 95% confidence levels. Total respiratory admission rates, including Influenza and pneumonia admissions were estimated for each age group and season by dividing the respiratory admissions by the population for each group. The influenza‐associated hospitalizations and rates per age group and season were then estimated and adjusted for the sensitivity and specificity of the SARI case definition using the following formula:

$$\text{Influenza}-\text{associated hospitalizations}=\left(\left(\text{positivity}+\text{specificity}-1\right)/\left(\text{sensitivity}+\text{specificity}-1\right)\right)*\text{total respiratory admission}$$

$$\text{Influenza}-\text{associated hospitalization rate}=\left(\left(\text{positivity}+\text{specificity}-1\right)/\left(\text{sensitivity}+\text{specificity}-1\right)\right)*\text{total respiratory admission rate}$$

For each season, average estimates were also computed.

### 2.5 Estimates by province of residence

Province‐specific frequencies and rates were estimated for each season. Cases with missing province of residence were excluded from the analysis. The difference in the coverage of the hospital billing database by province was taken into account in the calculation.^15^ Total respiratory admission rates including Influenza and pneumonia admissions were estimated for each province of residence and season by dividing the respiratory admissions (under J09‐J18 codes) by the population of each province of residence. The influenza‐associated hospitalizations and rates per province of residence and season were then estimated using positivity percentages for each season adjusting for the sensitivity and specificity of the SARI case definition as described above.

### 2.6 Burden of disease pyramid

The influenza disease burden pyramid tool was used to estimate influenza‐associated deaths and mild influenza illness. This tool uses a multiplier‐based approach to estimate the influenza disease burden across levels of severity using available data on influenza‐associated hospitalizations or deaths.^18^ For our study, the pyramid was generated using the estimated average age‐specific influenza‐associated hospitalizations over the five studied influenza seasons. In the tool, the mid‐year 2018 and the default multiplier were selected. Further, the tool was used with no corrections and no expansion factors. The average seasonal influenza counts by age strata across level of severity were estimated from the pyramid tool and rates were computed thereafter.

### 2.7 Ethical approval

Data received from all sources were anonymous. Further, the analysis in this study was based on surveillance data thus no ethical approval was needed.

## 3 RESULTS

Over the study period, a total of 45 801 hospitalizations with influenza/pneumonia diagnosis were extracted from the MoPH billing database with an average of 9160 per season ranging between 7034 in 2019–2020 and 11 271 in 2015–2016 (Table 1).

**TABLE 1:** Estimates of influenza‐associated respiratory hospitalization numbers and rates by season and age group, Lebanon, 2015–2020.

Season	Age group (y)	Respiratory admissions (J9–J18)^a^	Influenza positivity %(*n*/*N*)^b^	Estimated influenza‐associated respiratory hospitalizations	
				Count	Rate per 100 000 (95% CI)
2015–2016	0–4	3521	9.7(70/718)	455.82	81.5 (74.0–88.9)
	5–14	1210	15.6(29/186)	349.3	33.6 (30.0–37.1)
	15–49	2704	15.3(44/287)	761.5	23.2 (21.6–24.8)
	50–64	1705	13.0(19/146)	372.4	41.3 (37.1–45.5)
	≥65	2130	11.0(20/182)	347.7	73.4 (65.7–81.1)
	Total	11 271	12.0(182/1519)	2286.8	36.5 (35.0–38.0)
2016–2017	0–4	2647	5.6(33/589)	43.5	7.9 (5.6–10.3)
	5–14	857	8.0(11/138)	69.4	6.6 (5.0–8.1)
	15–49	1927	17.6(25/142)	662.1	21.2 (19.6–22.8)
	50–64	1474	12.1(12/99)	286.1	32.3 (28.5–36.0)
	≥65	2474	16.0(26/162)	745.1	150.8 (139.9–161.6)
	Total	9380	9.5(107/1130)	1806.3	29.6 (28.2–30.9)
2017–2018	0–4	2327	9.0(25/277)	255.3	48.1 (42.2–54.0)
	5–14	788	11.4(8/70)	138.1	12.9 (10.7–15.0)
	15–49	1656	11.9(17/143)	310.9	10.5 (9.3–11.7)
	50–64	1334	16.5(17/103)	418.4	48.0 (43.4–52.6)
	≥65	2154	17.4(25/144)	725.8	142.4 (132.0–152.7)
	Total	8262	12.5(92/737)	1848.5	31.1(29.6–32.5)
2018–2019	0–4	2713	20.1(63/313)	1118.7	222.4 (209.4–235.5)
	5–14	998	29.3(24/82)	660.2	60.3 (55.7–64.9)
	15–49	1976	23.9(44/184)	1018.7	36.3 (34.0–38.5)
	50–64	1544	24.1(27/112)	804.2	94.1 (87.6–100.6)
	≥65	2623	21.1(37/175)	1154.2	222.0 (209.2–234.8)
	Total	9854	22.5(195/866)	4756.0	82.3 (79.9–84.6)
2019–2020	0–4	1906	18.7(39/209)	709.7	150.2 (139.2–161.3)
	5–14	794	35.3(41/116)	656.8	59.5 (55.0–64.1)
	15–49	1441	30.9(55/178)	1017.3	37.6 (35.3–39.9)
	50–64	1071	33.0(29/88)	816.1	95.7 (89.2–102.3)
	≥65	1822	13.7(20/146)	432.0	81.6 (73.9–89.3)
	Total	7034	25.0(184/737)	3631.9	64.1 (62.0–66.2)

^a^ Based on MoPH hospital billing database.

^b^ Based on SARI sentinel surveillance database.

**FIGURE A1:**
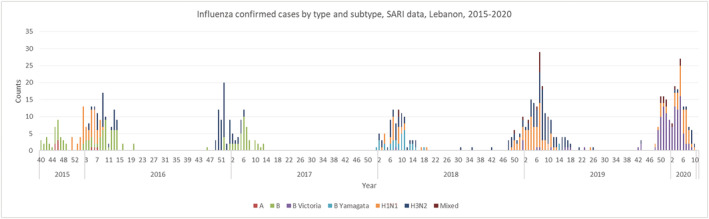
Influenza confirmed cases by type and subtype, SARI data, Lebanon, 2015–2020.

During the study period, a total of 4989 SARI cases were reported through the SARI sentinel surveillance system with a seasonal average of 997.8 cases, ranging between 737 in 2017–2018 and 2019–2020 and 1519 in 2015–2016. The distribution of influenza cases by time showed a seasonal pattern as shown in Figure A1 in Appendix A. As for the positivity during the study period, it was 16.3% ranging between 9.5% in 2016–2017 and 25.0% in 2019–2020 (Table 1).

The estimated seasonal average of the influenza‐associated respiratory hospitalizations was 2866 and for a rate of 48.1 per 100 000 (95% CI: 46.4–49.9) ranging between 29.6 per 100 000 (95% CI: 28.2–30.9) in 2016–2017 and 82.3 per 100 000 (95% CI: 79.9–84.6) in 2018–2019.

**FIGURE 1:**
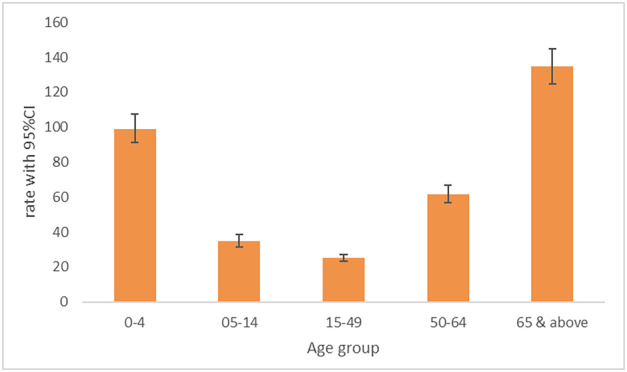
Average estimates of influenza‐associated respiratory hospitalization rates by age group, Lebanon, 2015–2020.

As for the distribution by age group, the highest rates were seen in the two age groups ≥65 years and 0–4 years while the lowest rate was for the age group 15–49 years. The average influenza‐associated hospitalization rates in the age group ≥65 years was 134.7 per 100 000 (95% CI:124.6–144.8) per season ranging between 73.38 per 100 000 (95% CI:65.67–81.09) in 2015–2016 and 222.00 per 100 000 (95% CI: 209.19–234.81) in 2018–2019. For the age group 0–4 years, it was 98.7 per 100 000 (95% CI:90.2–107.2) per season ranging between 7.90 per 100 000 (95% CI:5.55–10.25) in 2016–2017 and 222.44 per 100 000 (95% CI:209.40–235.47) in 2018–2019 (Table 1 and Figure 1).

**FIGURE 2:**
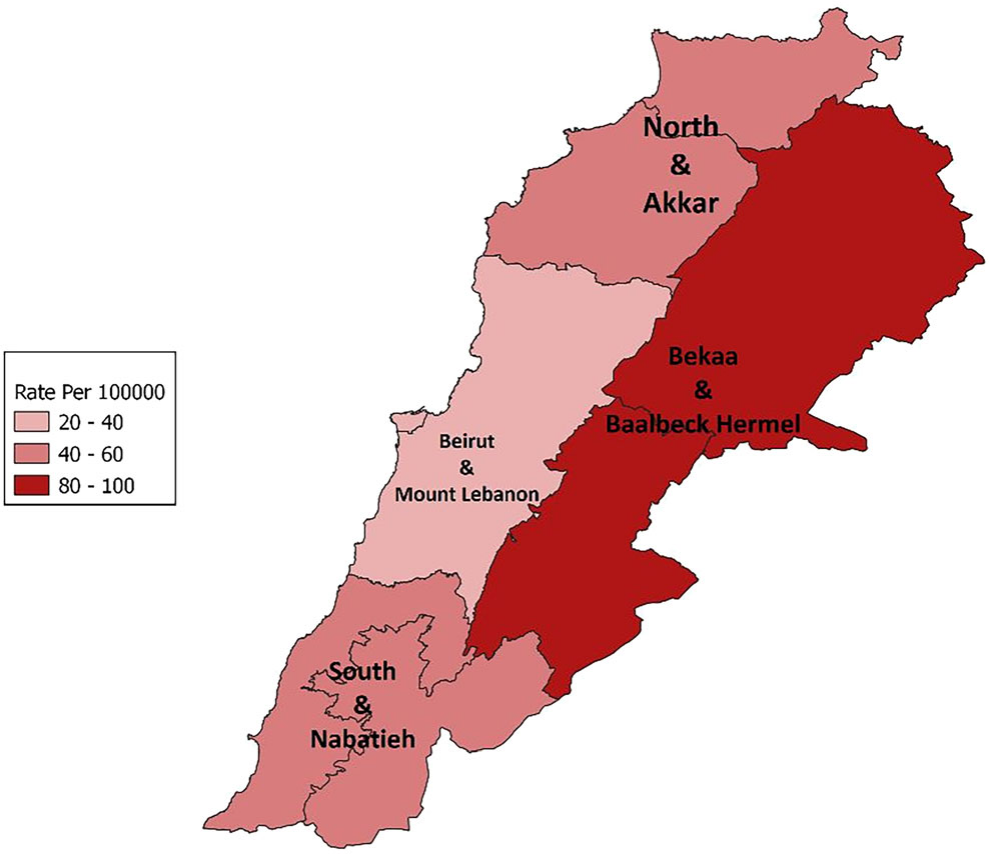
Estimates of seasonal average of influenza‐associated respiratory hospitalization rates by province of residence, Lebanon, 2015–2020.

As for the distribution by province of residence, the highest influenza‐associated respiratory hospitalization rates were reported from Bekaa and Baalback/Hermel provinces with a seasonal average of 82.9 per 100 000 (95% CI: 77.2–88.7) ranging between 31.05 per 100 000 (95% CI:27.61–34.48) in 2016–2017 and 155.80 per 100 000 (95% CI:147.84–163.76) in 2018–2019. The lowest rate was reported from Mount Lebanon and Beirut with an average of 45.5 per 100 000 (95% CI: 33.0–37.6) ranging between 14.12 per 100 000 (95% CI:12.69–15.54) in 2016–2017 and 64.82 per 100 000 (95% CI:61.68–67.96) (Table 2 and Figure 2).

**TABLE 2:** Estimates of influenza‐associated respiratory hospitalization numbers and rates by season and province of residence, Lebanon, 2015–2020.

Season	Governorate	Respiratory admissions (J9–J18)^a^	Estimated influenza‐associated respiratory hospitalizations	
			Count	Rate per 100 000 (95% CI)
2015–2016	Bekaa & Baalback/Hermel	3243	639.7	61.3 (56.6–66.1)
	Mount Lebanon &Beirut	2920	676.9	24.9 (23.0–26.8)
	North	2442	450.4	33.6 (30.5–36.7)
	South	2711	516.3	44.7 (40.8–48.6)
2016–2017	Bekaa & Baalback/Hermel	2472	313.5	31.1 (27.6–34.5)
	Mount Lebanon &Beirut	2526	376.5	14.1 (12.7–15.5)
	North	2136	253.2	19.4 (17.0–21.8)
	South	2201	269.5	23.9 (21.0–26.7)
2017–2018	Bekaa & Baalback/Hermel	2231	471.5	48.7 (44.3–53.1)
	Mount Lebanon &Beirut	2445	607.3	23.7 (21.8–25.6)
	North	1835	362.6	27.5 (24.7–30.3)
	South	1811	369.5	33.7 (30.2–37.1)
2018–2019	Bekaa & Baalback/Hermel	2984	1471.5	155.8 (147.8–163.8)
	Mount Lebanon &Beirut	2824	1636.7	64.8 (61.7–68.0)
	North	2216	1021.7	82.2 (77.20–87.28)
	South	1907	907.9	84.8 (79.3–90.4)
2019–2020	Bekaa & Baalback/Hermel	1926	1085.5	130.0 (122.2–137.7)
	Mount Lebanon &Beirut	1952	1292.9	51.2 (48.4–54.0)
	North	1540	811.5	69.5 (64.7–74.3)
	South	1496	814.0	71.7 (66.8–76.6)

^a^ Based on MoPH hospital billing database.

According to the burden of disease pyramid tool, influenza was estimated to cause 347 deaths each season in Lebanon mainly among adults ≥65 years old (239 deaths). The estimated seasonal number of critically ill cases among hospitalized patients was 770 cases ranging between 67 among children aged between 5 and 14 years old and 251 among adults ≥65 years old. It was estimated that influenza caused 977 448 mild/moderate cases each season mainly among those aged between 5 and 49 years old. On the other hand, the seasonal influenza‐associated mortality rate computed from the pyramid tool estimates was 5.83 per 100 000 for all age groups ranging between 0.96 among children less than 5 years old and 47.3 among adults ≥65 years old. The estimated rate of critically ill cases was 12.9 per 100 000 with the highest rates among adults ≥65 years old (49.7 per 100 000) and adults aged between 50 and 64 (20.8 per 100 000). Regarding the mild/moderate cases, the seasonal estimated rate was 16 420 per 100 000 with the highest rates among the age groups 5–14 and 15–49 (28 376 and 18 087 per 100 000, respectively) (Table 3).

**TABLE 3:** Estimates of seasonal influenza‐associated respiratory counts and rates by age group and severity level, Lebanon, 2015–2020.

Age group (y)	Counts				Rates (per 100 000)			
	Deaths	Critically ill	Hospitalized	Mild/moderate	Deaths	Critically ill	Hospitalized	Mild/moderate
0–4	5	78	517	75 902	0.96	14.9	98.8	14,505.5
5–14	11	67	375	304 772	1.02	6.2	34.9	28,375.7
15–49	50	192	754	538 385	1.7	6.5	25.3	18,087.3
50–64	42	182	539	52 444	4.8	20.8	61.7	6005.1
≥65	239	251	681	5945	47.3	49.7	134.7	1176.1
Total	347	770	2866	977 448	5.8	12.9	48.1	16,420.2

## 4 DISCUSSION

This is the first study conducted in Lebanon to estimate the burden of influenza at the national level. Influenza‐associated respiratory hospitalizations and rates were estimated by age group and province of residence. The influenza burden by level of severity was also estimated. The influenza‐associated respiratory hospitalization rate computed in this study for all age groups (48.1 per 100 000) is comparable to results of studies conducted in Egypt (44 per 100 000)^19^ and Zambia^20^ but higher than estimates of other countries like Canada (33 per 100 000), Portugal (19.4 per 100 000), Indonesia (13–19 per 100 000), Iran(29 per 100 000), and Oman(7.3 and 27.5 per 100 000).^9^, ^10^, ^11^, ^21^, ^22^ As for the estimated mortality rate for all age‐groups (5.8 per 100 000), it is comparable to the worldwide mortality estimate (5.9 per 100 000) but higher than estimates of the Eastern Mediterranean region (4.5 per 100 000).^23^

When comparing our study results to the country‐specific estimates of global studies, we find a lower estimated hospitalization rate in our study (48.1 per 100 000) compared with global estimates for Lebanon (145.9 per 100 000) and a higher mortality rate (5.8 per 100 000) compared with global estimates for Lebanon (0.6 per 100 000).^24^ In our study, we estimated that 347 influenza‐associated respiratory deaths occur each year (range: 11–720) in Lebanon. This is higher than the global country figures estimating the median seasonal influenza‐associated respiratory deaths to be 109 (95% CI: 40–399).^24^ Discrepancies between our study results and global estimates are due to the different approaches and data sources used in the two types of studies.

Differences in the burden of disease results between different studies are affected by the studied influenza seasons and the different approaches used, which depend on the available resources. In addition, some estimates are from high‐income countries, which might not be comparable to those from low and middle income countries due to the difference in age distribution, nutrition status, prevalence of high‐risk conditions, access to health care, and preventive measures mainly vaccination policies.^7^

According to our study results, the highest burden of influenza hospitalization is reported in the high‐risk groups mainly adults above 65 and children less than 5 years, which is consistent with other studies conducted in different countries in the world.^9^, ^10^, ^11^ These results could be used to feed into evidence‐based policies to increase vaccination uptake especially among high‐risk groups.

As for the distribution by province of residence, our study shows that the highest rates are reported from the Bekaa and Baalback/Hermel provinces that include around 16% of Lebanon's population and are known to be Lebanon's most important farming region.^25^ Further studies are needed to understand whether this difference is related to differential healthcare seeking behaviors or a different prevalence of risk factors for severe influenza across provinces.

As for the burden of disease pyramid, it helps in estimating the influenza‐associated deaths, critically ill cases, and mild/moderate non‐hospitalized cases. It is crucial to conduct further studies to estimate nonmedically attendant mild/moderate and death cases using surveillance data and compare influenza‐associated death estimates to mortality data collected by the national statistics department.

Our study has several limitations. The main limitation is related to estimating the total respiratory admissions in the country based on the MoPH billing database. It was assumed that all uninsured Lebanese who are eligible for MoPH coverage are benefiting from this type of insurance, which might not be a valid assumption. Moreover, population estimates by nationality were based on UNHCR data, which only include registered Syrian refugees and not counting unregistered Syrians living in the country. This might have led to overestimated rates. On the other hand, the selection of respiratory cases from the ICD‐10 codes related to influenza and pneumonia (J09‐J18) only could have underestimated the real burden of influenza as some cases might have been coded under J00‐J99 ICD‐10 codes or might be other consequences of the disease like cardiovascular events and exacerbation of comorbidities among others, which should not be ignored.^22^ On the other hand, the completeness of SARI sentinel surveillance data was not constant throughout the study period as some sites stopped reporting after 2018. Moreover, the 2019–2020 season ended sooner than other seasons due to COVID‐19 pandemic hence explaining the decrease in number of enrolled cases during these seasons.

## 5 CONCLUSION

This study shows the substantial burden of influenza in Lebanon mainly on high‐risk groups (≥65 years old and <5). It is crucial to translate these findings into policies and practices to reduce the burden of the disease. It is also crucial to conduct further studies to estimate the illness‐related expenditure and indirect costs in addition to the disease burden averted through vaccination mainly among high‐risk groups identified in this study. Further studies are also needed to determine the risk factors for severe outcomes associated with influenza illness as well as conducting knowledge, attitude, and practice (KAP) studies on the disease and vaccine hesitancy.

## AUTHOR CONTRIBUTIONS

Zeina Farah, Hala Abou El Naja, Stefano Tempia, and Nada Ghosn contributed to the design of the study and all authors contributed to the methodology. Zeina Farah did the data analysis and presentation of results. Zeina Farah, Hala Abou El Naja, and Stefano Tempia validated the study results. Zeina Farah and Hala Abou El Naja wrote the original draft of the manuscript. The work was supervised by Nadine Saleh, Patrick Maison, and Nada Ghosn. All authors reviewed and edited the final version of the manuscript.

## CONFLICT OF INTEREST STATEMENT

The authors have no conflict of interest to declare.

### PEER REVIEW

The peer review history for this article is available at https://www.webofscience.com/api/gateway/wos/peer-review/10.1111/irv.13138.

## Data Availability

The data that support the findings of this study are available from the corresponding author upon reasonable request.
